# Intrapartum and 30-Day Postpartum Complications in Patients With Antenatal COVID-19 Infection: A Retrospective Cohort Study

**DOI:** 10.1155/2024/5421129

**Published:** 2024-11-04

**Authors:** Sriya Donthi, Jacqueline Kloos, Kelly S. Gibson, Danielle Olson, David C. Kaelber

**Affiliations:** ^1^Case Western Reserve University School of Medicine, Cleveland, Ohio 44106, USA; ^2^Division of Maternal Fetal Medicine, Department of Obstetrics and Gynecology, MetroHealth, Cleveland, Ohio 44109, USA; ^3^Departments of Internal Medicine, Pediatrics, and Population and Quantitative Health Sciences, Case Western Reserve University, The Center for Clinical Informatics Research and Education, The MetroHealth System, Cleveland, Ohio 44106, USA

**Keywords:** COVID-19, pregnancy, SARS-CoV-2

## Abstract

**Objective:** The study was aimed at comparing intrapartum and postpartum outcomes between pregnant patients with and without antenatal COVID-19 infection using aggregated, deidentified electronic health record (EHR) data.

**Design and Setting:** This retrospective cohort study included data from over 80 health care organizations within the TriNetX Analytics Research Network.

**Population:** Individuals admitted for delivery from Jan 2020 to May 2023 were studied.

**Methods:** We studied individuals with ICD-10 codes for delivery, COVID-19 diagnosis, and primary outcomes. We compared the incidence of adverse intrapartum and 30-day postpartum outcomes in those with and without antenatal COVID-19.

**Main Outcome Measures:** The main outcomes compared were obstetric, cardiovascular, neurovascular, and respiratory outcomes within 30 days postpartum.

**Results:** Twenty-six thousand nine hundred seventy-four of 369,923 (7%) birthing parents with a delivery encounter had an antenatal COVID-19 diagnosis. Compared to matched controls, having COVID-19 was associated with an increased risk of postpartum hemorrhage (RR—1.24 (CI—1.16–1.33)), gestational hypertension (RR—1.27 (CI—1.27–1.34)), preeclampsia (RR—1.25 (CI—1.18–1.32)), eclampsia (RR—1.66 (CI—1.29–2.32)), preterm labor (RR—1.21 (CI—1.21–1.34)), cerebral infarction (RR—1.74 (CI—1.04–2.90)), cardiomyopathy (RR—2.08 (CI—1.30–3.32)), heart failure (RR—1.55 (CI—1.04–2.31)), sepsis (RR—2.21 (CI—1.54–3.19)), DVT (RR—2.32 (CI—1.45–3.71)), and pulmonary embolism (RR—2.68 (CI—1.74–2.90)).

**Conclusion:** Individuals with antenatal COVID-19 were more likely to have intrapartum and postpartum obstetric, cardiovascular, neurovascular, and respiratory complications. This data will inform risk stratification and screening for prenatal care providers.

## 1. Introduction

In March 2020, coronavirus disease 2019 (COVID-19) caused by SARS-CoV-2 infections was declared to be a global pandemic by the World Health Organization and affected 650 million individuals over the subsequent 2 years [1, 2]. COVID-19 infection has a known lasting impact and has been associated with a 12-month risk of incidental cardiovascular and respiratory complications in the general population [3]. Pregnancy renders individuals particularly susceptible to respiratory pathogens due to the physiological adaptations of decreased respiratory volumes, increased hypoxic tolerance, oxidative stress in the setting of increased metabolic demand, and immunomodulation [4, 5].

Previous small studies have reported varied associations between antenatal COVID-19 infection and maternal complications including hypertensive conditions, cardiomyopathy, cardiovascular events, thromboembolic events, sepsis, admission to the intensive care unit, preterm labor, cesarean sections, and maternal and neonatal mortality [4, 6–10]. Across the few larger studies that have evaluated antenatal COVID-19 infections and subsequent conditions, there were conflicting findings regarding the risk of complications such as hypertensive conditions of pregnancy and preterm labor [7, 11–14]. A small 2022 study looking at 86 pregnant women in the west Parisian area before and during the pandemic found no differences in rates of preeclampsia, placenta abruption, and stillbirth, while a 2020 study of 517 pregnant women between the short period of December 2019–May 2020 reported a 20% incidence of preeclampsia in the study population without comparison analyses [8, 10]. A between medium-sized study of 8239 pregnant patients between January 2020 and March 2021 found no increased risk of preeclampsia but did not report an increased incidence of eclampsia [11]. A study of 162, 576 pregnant patients conducted in Quebec, Canada, found elevated risks of acute renal failure, embolism, shock, sepsis, DIC, and hemorrhage but was limited by geography and subsequently by patient demographics [6]. One large US-based study including 1.6 million pregnant patients across 463 hospitals found small but significant increases in the risk of gestational hypertension (OR, 1.08), obstetrical hemorrhage (OR, 1.07), and preeclampsia (OR, 1.04), but the study design compared these patient outcomes before and during the pandemic (January 2020–March 2020) which limits the ability to associate outcomes with the SARS-CoV-2 infection [7]. A few meta-analyses including large population sizes from 10,000 to 400,000 pregnant patients with COVID-19 infection, limited by temporality of association between SARS-CoV-2 infections, did not report on maternal outcomes or reported increased risk of preeclampsia with severe features, eclampsia, and hemolysis, elevated liver enzymes, and low platelet (HELLP) syndrome, respectively [13, 14]. Overall, the inconsistent findings across current literature may be due to small sample sizes, variants of the virus that are excluded or overlap due to study time frames, study designs focused on temporality, and population demographics in studies limited to specific geographical regions.

This study is aimed at expanding on the current body of research evaluating the effects of antenatal COVID-19 infections by investigating whether individuals with COVID-19 infections during pregnancy are at higher risk of cardiovascular, pulmonary, infectious, and obstetric complications in the intrapartum and postpartum period. This study is aimed at addressing limitations seen in other studies by reporting on data from individual patient cases of COVID-19 infection studied across a broad time frame and across a much larger set of aggregated, deidentified electronic health record (EHR) data to include the various virus variants and patient demographics.

## 2. Methods

### 2.1. Study Design and Data Collection

**Figure 1:**
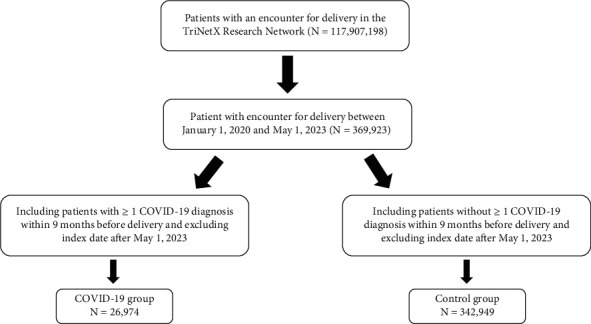
Flowchart of study and control populations sourced from the TriNetX Analytics Research Network.

The TriNetX Analytics Research Network platform (http://www.trinetx.com/), containing deidentified and aggregated EHR data across more than 80 health care organizations and 100 million patients, was used to conduct a retrospective cohort study from January 2020 to May 2023. Using TriNetX in the way described here has been deemed exempt from Institutional Review Board (IRB) review, approved by the Western IRB and the MetroHealth IRB, as well as expert attestation [15]. TriNetX queries were performed on May 1, 2023, using ICD-10 codes to define the study cohort.  Figure 1 describes the study and control populations sourced from the TriNetX Analytics Research Network. This study and report conform to the STROBE criteria [16]. Relevant core outcome sets do not exist currently for the maternal nonobstetrical complications studied here. This study did not include accessing any individual patient data.

### 2.2. Study Population

This study population includes all individuals of all ages who had an encounter diagnosis for delivery defined by ICD-10 Code O80-O82 between January 1, 2020, and May 1, 2023, which includes the defined COVID-19 pandemic time period per the World Health Organization [2, 17]. Patients were determined to have an antenatal COVID-19 infection if they have an encounter diagnosis for delivery (ICD-10 Code O80-O82) and at least one encounter diagnosis for COVID-19 (identified via ICD-10 Code U70.1) occurring within 9 months prior to encounter diagnoses for delivery. These patients were grouped into the study group with antenatal COVID-19 infection. Patients in the control group were defined as those with one encounter diagnosis for delivery (ICD-10 Code O80-O82) and without any encounter diagnoses for COVID-19 (identified via ICD-10 Code U70.1) occurring within 9 months prior to encounter diagnosis for delivery. Those with pre-existing encounter diagnoses ICD10 codes of primary outcomes were not excluded. Patients in both the antenatal COVID-19-negative and antenatal COVID-19-positive cohorts are assumed to have the same rate of pre-existing diagnoses in the general population. The control and COVID-19 infection groups were propensity matched for age, race, and ethnicity. Data in TriNetX updates regularly, so analysis should typically occur in as short a time frame as possible. As a result, for this study, TriNetX queries and subsequent analyses were performed once on May 1st, 2023.

### 2.3. Main Outcomes and Definitions

Outcomes that occurred on the day of delivery through 30 days postpartum were included. It is well established that the viral infections increase the physiological susceptibility of the cardiorespiratory system and the immune system [2, 17].This study includes outcomes that occur as a result of the involvement of these systems, including cardiovascular (acute myocardial infarction and cardiomyopathy) or venous conditions (venous thromboembolism events) and sepsis. Moreover, animal models have shown increased expression and activity of the main receptor for the SARS-CoV-2 virus, angiotensin-converting enzyme 2 (ACE2), in the kidney, uterus, and placenta during pregnancy [9, 13]. As a result, this study focuses on outcomes that can occur from the involvement of these organs, including hypertensive disorders of pregnancy, hemorrhage, and sepsis. Moreover, prior studies on maternal outcomes have also reported on these outcomes related to increased physiological susceptibility during/after viral infection. Rarer complications, such as maternal death and abortive outcomes, were not compared due to the low incidence of these outcomes. These outcomes, identified by ICD-10 codes, were defined as follows: cerebral infarction (I63), pulmonary embolism (I26), acute embolism and thrombosis of deep veins of the lower extremity (I82.4), sepsis (A41), heart failure (I50), acute myocardial infarction (I21), cardiomyopathy (I42), placental abruption (O45), postpartum hemorrhage (O72), preterm labor (O60), gestational hypertension without significant proteinuria (O13), preeclampsia (O14), HELLP syndrome (O14.2), and eclampsia (O.15). Encounter for delivery was defined by ICD-10 Code O80-O82, and COVID-19 infection was defined by ICD-10 Code U07.1.

### 2.4. Statistical Analysis

A 95% confidence interval (CI) was used to calculate relative risk. Demographic data was analyzed using two-sample *Z*-tests for continuous variables and chi-square tests for categorical variables (*p* < 0.05). To preserve statistical deidentification in TriNetX, any patient counts of 1–10 are reported at 10. These patient counts are depicted with superscript letters in our tables. The statistical analysis was performed using analytic tools built into the TriNetX platform. Attempted analysis of COVID-19 infection by trimester was limited by low patient counts.

## 3. Results

Prior to propensity matching, our search criteria produced 342,949 patients without antenatal COVID-19 infections and 26,974 patients with antenatal COVID-19 infections. Propensity matching was conducted within the TriNetX platform and controlled for age, race, ethnicity, and comorbidities documented within 270 days prior to delivery including cerebral infarction, pulmonary embolism, other venous embolism/thrombosis, sepsis, heart failure, cardiomyopathy, preterm labor, premature rupture of membrane, postpartum hemorrhage, placental abruption, gestational hypertension, preeclampsia, eclampsia, and acute myocardial infarction. After propensity matching, both groups contained 26,974 patients matched for age, race, ethnicity, and comorbidities. The average age at the time of delivery was 29.9 ± 7.26 and 29.9 ± 7.17 years in the antenatal COVID-19 infection group and no antenatal COVID-19 infection group, respectively.  Table 1 shows the demographic characteristics of the participants in both cohorts.

Individuals with antenatal COVID-19 infections were found to have an increased risk of several cardiovascular, pulmonary, infectious, and obstetric complications within 30 days of delivery (Table 2). Table 2 shows the incidence of adverse intrapartum and postpartum outcomes among the two cohorts. Those with COVID-19 infection during pregnancy had increased risk of cerebral infarction (RR—1.74, 95% CI—1.04–2.90), pulmonary embolism (RR—2.68, 95% CI—1.74–4.13), acute embolism and thrombosis of deep veins (RR—2.32, 95% CI—1.45–3.71), sepsis (RR—2.21, 95% CI—1.54–3.19), heart failure (RR—1.55, 95% CI—1.04–2.31), and cardiomyopathy (RR—2.08, 95% CI—1.30–3.32) (Table 2). We were unable to adequately assess the relative risk of acute myocardial infarction given its low prevalence.

Pregnant individuals with COVID-19 infections during their pregnancy were found to have increased prevalence of postpartum hemorrhage (RR—1.24, 95% CI—1.16–1.33) and an increased risk of gestational hypertension (RR—1.27, 95% CI—1.21–1.34), preeclampsia (RR—1.25, 95% CI—1.18–1.32), and eclampsia (RR—1.66, 95% CI—1.29–2.23) from the day of delivery to 30 days postpartum (Table 2). We also considered several intrapartum complications and found increased risk for preterm labor (RR—1.21, 95% CI—1.12–1.31) (Table 2). There was no statistically significant increased risk observed in the prevalence of placental abruption (RR—1.14, 95% CI—0.94–1.37) or of HELLP syndrome (RR—1.18, 95% CI—0.96–1.44) between the two cohorts.

## 4. Discussion

### 4.1. Main Findings

This study utilized aggregated EHR data from across the globe (~80% from the US) to report a comparison of adverse intrapartum and postpartum outcomes in individuals who were and were not infected by COVID-19 during the antenatal period. Controlling for age, race, and ethnicity, patients with COVID-19 infections during pregnancy were at an increased risk for several cardiovascular, pulmonary, infectious, and obstetric complications from the day of delivery through the 30-day postpartum period. Patients with antenatal infection were more than twice as likely to develop a pulmonary embolism, deep vein thrombosis, sepsis, and cardiomyopathy in the 30-day postpartum period. They were also more likely to develop cerebral infarctions or heart failure in this interval. This cohort was also at a slightly increased risk of postpartum hemorrhage and the elevated blood pressure syndromes ranging from gestational hypertension without proteinuria to eclampsia, with the exception of HELLP syndrome, during the 30-day postpartum period.

### 4.2. Strengths and Limitations

To our knowledge, this is the largest study examining intrapartum and postpartum outcomes in patients with antenatal COVID-19 infection [18, 19]. There are conflicting reports in previous studies regarding the risk of obstetric and nonobstetric complications in such patients [5, 7–14]. This variation may be due to smaller sample sizes, timing of data collection during the pandemic, or timing of the infection during gestation. Our study is notable for its size and generalizability across populations, including data globally from the start to the end of the pandemic [2, 17].

However, our analysis is not without its limitations. The use of ICD-10 codes within an aggregated EHR platform assumes that all encounter diagnoses are documented in the EHRs of contributing health care organizations. It is possible that COVID-19 encounter diagnoses were not recorded in the EHR due to home testing, lack of testing, or lack of reporting. Undiagnosed and unreported COVID-19 encounter diagnoses, as part of our control group, would impact the estimated risk of COVID-19-affiliated complications. Additionally, we could not obtain the prior infection or vaccination status of our cohort. It is possible that vaccinated or previously exposed patients who then experienced antenatal infection were at a decreased risk for complications, underestimating the true risk in previously COVID-19-naïve individuals. This limitation could be partially mitigated in future research by excluding vaccinated participants or only including those who delivered prior to the COVID-19 vaccine. However, both strategies would decrease sample size, and the former would require documentation of vaccination in the EHR. Finally, the relative risk of acute myocardial infarction could not be calculated accurately given that TriNetX reports results of 1-10 patients as 10 patients to present statistical de-identified.”To represent most accurate explanation. This is done to preserve anonymity but can also lead to overreporting of diagnosis numbers and overestimation of prevalence. This study contains data from 2020 to 2023, during which multiple strains of the SARS-CoV-2 virus emerged, for which distinct ICD-10 codes are not specified. The conclusions are unable to identify which strains of the virus are correlated to the rates of a given primary outcome.

### 4.3. Interpretation

Our data shows an increased risk of cardiac complications, including cardiomyopathy and heart failure, on the day of delivery or within 30 days postpartum in those with antenatal COVID-19 infections. The pathophysiology of COVID-19-induced cardiovascular disease is multifactorial and relates to electrolyte imbalances, endothelial inflammation, direct infection, fever, cytokine storm, and hypoxemia [20]. The SARS-CoV-2 infection is known to involve the ACE2 receptor, found in the cardiovascular system, and can lead to both direct infection or secondary involvement of the myocardial tissue [21]. Our data shows that maternal complications and outcomes resulting from direct infection had significantly increased risk in patients with antenatal COVID-19 infections compared to outcomes resulting from secondary involvement (acute myocardial infarction [RR 1.40, 95% CI 0.62–3.15]). Heart failure (RR 1.55, 95% CI 1.04–2.32) and cardiomyopathy (RR 2.08, 95% CI 1.30–3.32) are postulated to be seen in those with COVID-19 infections secondary to direct insult via the ACE2 receptor found on the myocardial and cardiovascular tissue or due to hypoxia and acute respiratory distress syndrome–induced pulmonary hypertension, which may be further exacerbated in the pregnant population due to decreased respiratory volumes and the edematous respiratory tracts seen in many viral infections [4, 21].

Our data shows an increased risk of pulmonary embolism, cerebral infarction, acute embolism, and thrombosis of deep veins of the lower extremity in those with antenatal COVID-19 infection. These findings are consistent with previous studies [4, 9]. An increased risk of thrombotic events in the pregnant population is known due to underlying hemostatic changes that occur to support pregnancy and prevent hemorrhage. SARS-CoV-2 infection can cause cytokine storm which further activates coagulation pathways [22, 23]. The severe inflammatory cytokine responses and endothelial damage generated in a COVID-19 infection exacerbate the hypercoagulable state of pregnancy, which can further contribute to thrombotic and embolic events [21–23]. The increased risk of thrombotic events can translate into clinical recommendations to include a higher degree of suspicion for thrombosis in pregnant patients with COVID-19 infection during pregnancy and to consider a lower threshold for obtaining diagnostic studies and considering anticoagulation. For patients with COVID-19 severe enough to warrant admission, the majority will have a Caprini score warranting anticoagulation [24]. In the absence of VTE diagnosis or other clinically significant risk factors, there is not sufficient evidence to support the use of ambulatory anticoagulation.

Our data also shows an increased risk of intrapartum and 30-day postpartum sepsis in patients with antenatal COVID-19 infections. Prior studies vary, with one finding stable rates of sepsis when comparing the prepandemic and pandemic periods [7] and others reporting increased risk of maternal sepsis in COVID-19-positive patients [10, 23]. Sepsis may be secondary to viral or bacterial infections that are superimposed secondary to mucosal and respiratory microbiome changes and immunomodulatory effects of pregnancy [9].

With regard to obstetric complications, our data shows that antenatal COVID-19 infections are associated with an increased risk of preterm birth, several gestation hypertensive diseases, and postpartum hemorrhage. These findings support prior studies that reported that preterm birth was more likely in individuals with COVID-19 infection during pregnancy than in those without [4, 13, 23]. Hypoxemia and acidosis, which can both be seen in COVID-19 infections, are both independent risk factors for preterm labor [4].

With regard to obstetric hypertensive diseases, our data supports prior findings that report an increased risk of eclampsia and preeclampsia [24, 25]. This may be secondary to the breakdown of maternal–fetal immune tolerance, endothelial dysfunction, and uteroplacental malperfusion and ischemia seen in SARS-CoV-2 infection [24]. SARS-CoV-2 can infect endothelial cells via ACE2 receptors, present in the placenta, and lead to microvascular damage in the same way that syncytiotrophoblast stress typically leads to endothelial damage in patients without COVID-19 infection [25]. There have been reports of a dose-dependent relationship between the severity of the SARS-CoV-2 infection by symptom severity (per NIH clinical spectrum of SARS-CoV-2 infection) and the development of preeclampsia [24]. It is known that phenotypes of COVID-19 and preeclampsia with severe features can overlap. Significant COVID-19 infections early in pregnancy seem to be associated with growth abnormalities and other markers of placental insufficiency later in pregnancy [24]. However, the mechanism for these associations is unknown. ACE2 expression variation may play a role in the development of preeclampsia in those with antenatal COVID-19 infection, but it is likely more multifactorial.

In contrast to other studies that found an increased rate of development of HELLP syndrome in those with SARS-CoV-2 infections [13, 14], our data found that HELLP syndrome occurred at the same rate in those with and without antenatal COVID-19 infections. While biomarker data was not used in this study, future studies can incorporate biomarker data on endothelial dysfunction and inflammation to further elucidate the underlying pathophysiological mechanism that associates COVID-19 infection with hypertensive disorders of pregnancy. Research regarding biomarkers and preeclampsia is ongoing and focused on potential sFlt-1/PlGF ratios, and continued evaluation of biomarkers may add to the current understanding of the correlation between COVID and hypertensive processes of pregnancy [4, 25]. We suggest that the risks of hypertensive complications in patients with antenatal COVID-19 infection can be mitigated via increased screening (inquiring if patients were affected by COVID-19 during their pregnancy), increased patient education that antenatal infection may increase the risk of hypertensive disorders of pregnancy, and increased surveillance of blood pressures and symptoms in those who develop antenatal infection. While there is no data to support antenatal surveillance in the absence of fetal growth abnormalities, hypertensive processes and placental dysfunction are associated and lagging fetal growth can be used as a marker for developing placental insufficiency.

Our results also found that patients with COVID-19 infection had an increased risk of postpartum hemorrhage on the day of delivery or within the 30-day postpartum period, which may be explained by the SARS-CoV-2-induced cytokine storm altering hemostatic capabilities [24]. However, we found that placental abruption occurred at the same rate in those with and without antenatal COVID-19 infections, a deviation from the increased risk previously observed with acute upper respiratory diseases and pneumonia [25, 26]. ACE2 expression by the placenta may allow viral entry, but some reports claim that the placenta provides both a structural and immunologic defense against COVID-19 [27]. Future studies can examine the development of various placental pathologies in pregnant patients with COVID-19 infections.

The development and inclusion of maternal neurovascular, respiratory, and infectious intrapartum and postpartum complications as core outcomes in future studies would assist in exploring COVID-19 infection as a maternal risk factor.

## 5. Conclusions

We found a significantly increased risk of cardiovascular, respiratory, infectious, and obstetric complications during the 30-day postpartum time period in birthing parents with COVID-19 infection during pregnancy after controlling for age, race, and ethnicity. Prior exploration on this topic has produced conflicting results regarding the risk of maternal complications and was limited by sample size, decreased generalizability due to narrow patient demographics and geographical restrictions, short time periods of study that exclude/overlap viral strains, and temporality. This study addresses these limitations and provides more definitive results by using a large global dataset to report on data collected over a longer time period to account for various viral strains and the rate of complication/outcome development in the setting of a pregnancy course. This large study indicates that a higher degree of suspicion for serious illness should be afforded to recently pregnant individuals with a history of antenatal COVID-19 infection and may emphasize the importance of vaccination. Obstetricians and health care providers should approach their monitoring and management of patients with antenatal COVID-19 infection with a heightened awareness of the hypertensive risks and complications that this patient population may develop during the intrapartum and immediate postpartum period. Antenatal screening, thorough assessment of historical COVID-19 symptoms or positive home test results, patient education, and improved surveillance of blood pressures and related symptoms through pregnancy in those who develop antenatal COVID-19 infection can help mitigate the risks of hypertensive complications through detection and awareness of COVID-19 infection as a risk factor.

## Figures and Tables

**Table 1:** Demographic characteristics for patients with and without gestational COVID-19 infections prior to and after propensity matching for age, race, and ethnicity.

**Population characteristic**	**Prior to propensity matching for age, sex, and ethnicity**		**After propensity matching for age, sex, and ethnicity**	
	**Patients with antenatal COVID-19 infections** **26974**	**Patients without antenatal COVID-19 infections** **342,949**	**Patients with antenatal COVID-19 infections** **26974**	**Patients without antenatal COVID-19 infections** **26974**
Age at delivery, mean (SD)	29.9 (7.26)	29.6 (7.03)	29.9 (7.26)	29.9 (7.17)
Race, *n* (%)				
White	16,109 (60)	178,467 (52)	16,108 (60)	16,208 (60)
Unknown	5489 (20)	98,289 (28)	5424 (20)	5513 (20)
Black or African American	4286 (16)	51,853 (16)	4286 (16)	4228 (16)
Asian	905 (3)	12,717 (4)	904 (3)	918 (3)
American Indian or Alaska Native	86 (0)	1001 (0)	86 (0)	57 (0)
Native Hawaiian or Other Pacific Islander	65 (0)	981 (0)	67 (0)	70 (0)
Ethnicity (% of cohort)				
Unknown	4985 (18)	100,498 (29)	4985 (18)	4872 (18)
Not Hispanic or Latino	16,109 (59)	178,467 (52)	15,978 (59)	16,136 (60)
Hispanic or Latino	5879 (22)	63,847 (19)	5880 (22)	5893 (22)

**Table 2:** Comparison of outcomes within 30 days of delivery in patients with and without COVID-19 during pregnancy matched by age, race, and ethnicity.

**Outcome (ICD-10 code)**	**Percent prevalence of 30-day postpartum outcome in patients with antenatal COVID-19 infection (%)**	**Percent prevalence of 30-day postpartum outcome in patients without antenatal COVID-19 infection (%)**	**Relative risk (95% CI)**	**Significance (** **p** ** value)**
Cerebral infarction (I63)	40/26,974 (0.148%)	23/26,974 (0.085%)	1.74 (1.04–2.90)	0.0321
Pulmonary embolism (I26)	75/26,974 (0.278%)	28/26,974 (0.104%)	2.68 (1.74–4.13)	< 0.0001
Acute embolism and thrombosis of deep veins of lower extremity (I82.4)	58/26,974 (0.215%)	25/26,974 (0.093%)	2.32 (1.45–3.71)	0.0003
Sepsis (A41)	93/26,974 (0.345%)	42/26,974 (0.156%)	2.21 (1.54–3.19)	< 0.0001
Heart failure (I50)	62/26,974 (0.23%)	40/26,974 (0.148%)	1.55 (1.04–2.31)	0.0292
Acute myocardial infarction	14/26,974 (0.052%)	10/226,974 (0.037%)^a^	1.4 (0.62–3.15)^a^	0.4141
Cardiomyopathy	54/26,974 (0.2%)	26/26,974 (0.096%)	2.08 (1.30–3.32)	0.0017
Placental abruption (O45)	227/26,974 (0.842%)	200/26,974 (0.741%)	1.14 (0.94–1.37)	0.1896
Postpartum hemorrhage (O72)	1728/26,974 (6.406%)	1391/26,974 (5.157%)	1.24 (1.16–1.33)	< 0.0001
Preterm labor (O60)	1224/26,974 (4.538%)	1011/26,974 (3.748%)	1.21 (1.12–1.31)	< 0.0001
Gestational hypertension without significant proteinuria	2895/26,974 (10.733%)	2280/26,974 (8.453%)	1.27 (1.21–1.34)	< 0.0001
Preeclampsia	2398/26,974 (8.89%)	1924/26,974 (7.133%)	1.25 (1.18–1.32)	< 0.0001
HELLP syndrome	201/26,974 (0.745%)	171/26,974 (0.634%)	1.18 (0.96–1.44)	0.1186
Eclampsia	139/26,974 (0.515%)	82/26,974 (0.304%)	1.66 (1.29–2.23)	0.0001

^a^Diagnoses with at least 1 but fewer than 11 patients.

## Data Availability

All data obtained for this study can be found on the TriNetX Analytics Research Network platform (http://www.trinetx.com/).
